# Metagenomic Next-Generation Sequencing of the 2014 Ebola Virus Disease Outbreak in the Democratic Republic of the Congo

**DOI:** 10.1128/JCM.00827-19

**Published:** 2019-08-26

**Authors:** Tony Li, Placide Mbala-Kingebeni, Samia N. Naccache, Julien Thézé, Jerome Bouquet, Scot Federman, Sneha Somasekar, Guixia Yu, Claudia Sanchez-San Martin, Asmeeta Achari, Bradley S. Schneider, Anne W. Rimoin, Andrew Rambaut, Justus Nsio, Prime Mulembakani, Steve Ahuka-Mundeke, Jimmy Kapetshi, Oliver G. Pybus, Jean-Jacques Muyembe-Tamfum, Charles Y. Chiu

**Affiliations:** a Department of Laboratory Medicine, University of California, San Francisco, San Francisco, California, USA; b UCSF-Abbott Viral Diagnostics and Discovery Center, University of California, San Francisco, San Francisco, California, USA; c Institut National de Recherche Biomédicale, Kinshasa, Democratic Republic of the Congo; d Department of Zoology, University of Oxford, Oxford, United Kingdom; e Etiologic, Inc., San Francisco, California, USA; f Department of Epidemiology, School of Public Health, University of California, Los Angeles, Los Angeles, California, USA; g Institute of Evolutionary Biology, University of Edinburgh, Edinburgh, United Kingdom; h Ministry of Public Health, Kinshasa, Democratic Republic of the Congo; i Department of Medicine, Division of Infectious Diseases, University of California, San Francisco, San Francisco, California, USA; Rhode Island Hospital

**Keywords:** 2014 Boende outbreak, Orungo virus, coinfection, Ebola virus, molecular clock analysis, next-generation sequencing, pathogen discovery, phylogenetic analysis, viral genome assembly, viral metagenomics

## Abstract

We applied metagenomic next-generation sequencing (mNGS) to detect Zaire Ebola virus (EBOV) and other potential pathogens from whole-blood samples from 70 patients with suspected Ebola hemorrhagic fever during a 2014 outbreak in Boende, Democratic Republic of the Congo (DRC) and correlated these findings with clinical symptoms. Twenty of 31 patients (64.5%) tested in Kinshasa, DRC, were EBOV positive by quantitative reverse transcriptase PCR (qRT-PCR). Despite partial degradation of sample RNA during shipping and handling, mNGS followed by EBOV-specific capture probe enrichment in a U.S. genomics laboratory identified EBOV reads in 22 of 70 samples (31.4%) versus in 21 of 70 (30.0%) EBOV-positive samples by repeat qRT-PCR (overall concordance = 87.1%). Reads from Plasmodium falciparum (malaria) were detected in 21 patients, of which at least 9 (42.9%) were coinfected with EBOV. Other positive viral detections included hepatitis B virus (*n* = 2), human pegivirus 1 (*n* = 2), Epstein-Barr virus (*n* = 9), and Orungo virus (*n* = 1), a virus in the *Reoviridae* family. The patient with Orungo virus infection presented with an acute febrile illness and died rapidly from massive hemorrhage and dehydration. Although the patient’s blood sample was negative by EBOV qRT-PCR testing, identification of viral reads by mNGS confirmed the presence of EBOV coinfection. In total, 9 new EBOV genomes (3 complete genomes, and an additional 6 ≥50% complete) were assembled. Relaxed molecular clock phylogenetic analysis demonstrated a molecular evolutionary rate for the Boende strain 4 to 10× slower than that of other Ebola lineages. These results demonstrate the utility of mNGS in broad-based pathogen detection and outbreak surveillance.

## Abstract (precis)

We applied metagenomic next-generation sequencing (mNGS) to detect Zaire Ebola virus (EBOV) and other potential pathogens from whole-blood samples from 70 patients with suspected Ebola hemorrhagic fever during a 2014 outbreak in Boende, Democratic Republic of the Congo (DRC) and correlated these findings with clinical symptoms. Twenty of 31 patients (64.5%) tested in Kinshasa, DRC, were EBOV positive by quantitative reverse transcriptase PCR (qRT-PCR).

## INTRODUCTION

Ebola virus (EBOV) is an infectious RNA filovirus primarily transmitted to humans by close contact with body fluids from infected patients or animals and consists of 5 species, including the prototype Zaire ebolavirus (EBOV) strain discovered in 1976 (1). Ebola virus disease (EVD), often fatal in its most severe manifestation of viral hemorrhagic fever, has remained a major public health concern in many parts of sub-Saharan Africa since its first appearance in 1976 in Zaire (now the Democratic Republic of the Congo [DRC]) (2). Symptoms of EVD include sudden onset of fever, muscle pain, headache, and sore throat, followed by vomiting, diarrhea, rash, and both internal and external bleeding (e.g., blood in stools and bleeding in the gums) (3). In addition to EVD, viral hemorrhagic fever has been associated with a range of pathogens, including flaviviruses (yellow fever and dengue virus), arenaviruses (Lassa fever), bunyaviruses (Rift Valley fever and Crimean-Congo hemorrhagic fever virus), and other filoviruses (Marburg virus) (4).

Between December 2013 and January 2016, West Africa, particularly, Guinea, Liberia, and Sierra Leone, experienced the largest EVD epidemic in history. More than 28,000 people were infected with EBOV, with more than 11,000 people dying from the disease (5). While the world focused on the West Africa outbreak, the World Health Organization (WHO) was notified of a separate but concurrent outbreak in the vicinity of Boende town, Équateur province, located in western Democratic Republic of the Congo (formerly Zaire). The index case was recorded on 26 July 2014 in a pregnant woman married to a bushmeat hunter living in Inkanamongo village, close to the town of Boende (6). The 2014 Boende outbreak marked the seventh Ebola outbreak in the Democratic Republic of the Congo since the discovery of the virus in 1976 and ended by October 2014. A previous analysis of the 2014 Boende outbreak reported a total of 69 patients diagnosed with suspected, probable, or confirmed EVD of 128 screened, with 49 (71.0%) deaths (6). Several reasons have been proposed for the significantly smaller size of the Boende outbreak (and other outbreaks in the Democratic Republic of the Congo) compared to the large epidemic that occurred in West Africa around the same time. These include the remote and isolated location of the Boende area, limiting the number of human contacts and potential exposure of the population, and the quick and effective responses by DRC public health agencies following the 6 previous EVD epidemics in the country.

In this study, we applied metagenomic next-generation sequencing (mNGS) as a tool for pathogen detection and genomic surveillance to identify EBOV and other infections in whole-blood samples obtained from patients during the 2014 Boende outbreak. In addition, we performed molecular clock and phylogenetic analyses of EBOV genomes to reconstruct the evolution of the 2014 Boende outbreak strain and its relationship to previous outbreak lineages.

## MATERIALS AND METHODS

### Ethics, consent, and permissions.

This study was approved by the Ministry of Health in the Democratic Republic of the Congo. Patients (*n* = 70) were enrolled from 13 August 2014 to 8 September 2014, during the middle of the 2014 Boende epidemic, and provided oral consent for enrollment in the study and collection and analysis of their blood. Consent was obtained at the homes of patients or in hospital isolation wards by a team that included staff members of the Ministry of Health. Coded whole-blood samples were analyzed at University of California, San Francisco (UCSF) under a protocol approved by the Institutional Review Board (protocol 11-05519).

### Sample collection and case definitions.

Epidemiologic and clinical data were collected using the World Health Organization (WHO) clinical investigation form for viral hemorrhagic fever according to standard case definitions (7). Samples from suspected cases were independently assayed for EBOV infection using up to 3 different molecular tests: (i) EBOV quantitative reverse transcriptase PCR (qRT-PCR_DRC_) performed at Institut National de Recherche Biomédicale (INRB), the national reference laboratory for viral hemorrhagic fever in Kinshasa, DRC (8), (ii) EBOV qRT-PCR (qRT-PCR_US_) performed subsequently at UCSF after transfer to the United States (9), and (iii) mNGS followed by EBOV probe enrichment, performed at UCSF in parallel with the qRT-PCR_US_ testing. Confirmed EVD cases (“confirmed EVD”) were defined as positive by at least 2 of the 3 molecular tests, whereas probable EVD cases (“probable EVD”) were defined as positive by a single test. Given sample degradation during shipment to the United States (see below), cases negative by all 3 molecular tests were defined as EBOV negative (“non-EVD”) if qRT-PCR_DRC_ testing had been performed; otherwise, they were classified as indeterminate (“indeterminate EVD”).

### Nucleic acid extraction.

Whole-blood samples were subjected to total nucleic acid extraction using the QIAamp viral RNA kit (Qiagen) at INRB in the Democratic Republic of the Congo. Following extraction, RNA was preserved using RNAstable (Biomatrica, Inc.) and shipped at room temperature to UCSF for metagenomic sequencing and PCR analysis. Partial degradation of the RNA occurred during shipment, as the RNAstable matrix was inadvertently not fully dried prior to shipment per the manufacturer’s recommendations. Upon receipt, RNA samples were resuspended in 20 μl water. RNA integrity was assessed using the Agilent Bioanalyzer RNA 6000 Pico kit.

### qRT-PCR_DRC_ EBOV assay.

The qRT-PCR_DRC_ EBOV assay, run in the Democratic Republic of the Congo, was performed as previously described (8). Briefly, qRT-PCR was performed using the LightCycler 480 RNA Master Hydrolysis Probes kit (Roche) by adding 5 μl of RNA to 20 μl of master mix containing 9.25 μl of reaction buffer, 1.6 μl of activator, 1 μl of enhancer, 7.85 μl of nuclease-free water, and 0.3 μl of a mix of primers and probes targeting the EBOV polymerase (L) gene (EBOVLF, 5′-GCGCCGAAGACAATGCA; EBOVLR, 5′-CCACAGGCACTTGTAACTTTTGC; EBOVLP, 5′-6FAM-TGGCCGCCAGCCT-MGBNFQ). The qRT-PCR assay was run on a SmartCycler (Cepheid) real-time PCR instrument using the following cycling conditions: 61°C for 300 s, 95°C for 30 s, followed by 45 cycles of 95°C for 15 s and 60°C for 40 s, with a fluorescence measurement at the end of each cycle. A threshold cycle (*C_T_*) value of 41 or less was considered positive.

### qRT-PCR_US_ EBOV assay.

The qRT-PCR_US_ EBOV assay, run in the United States, was performed as previously described (9). Briefly, qRT-PCR was performed using a Stratagene MX300P real-time PCR instrument and the QuantiTect reverse transcription kit (Qiagen) in a 25-μl total reaction volume (6.25 μl 2× QuantiScript, 0.125 μl of reverse transcriptase, 1 μl sample extract), with 0.125 μM each primer (F565, 5′-TCTGACATGGATTACCACAAGATC-3′; R640, 5′-GGATGACTCTTTGCCGAACAATC-3′). Conditions for the qRT-PCR were modified as follows: 50°C for 30 min and 95°C for 15 min, followed by 40 cycles of 95°C for 15 s, 57°C for 30 s, and 72°C for 30 s, with a fluorescence measurement at the end of each cycle. EBOV loads in genome copies per milliliter of sample were determined using standard curve analysis of an EBOV amplicon (see Fig. S1 in the supplemental material).

### RT-PCR confirmation by PCR and Sanger sequencing.

Confirmatory RT-PCR assays were performed using the Qiagen one-step RT-PCR kit in a 25-μl reaction volume. Conditions for the RT-PCR were as follows: 50°C for 30 min and 95°C for 15 min, followed by 40 cycles of 95°C for 30 s, 54°C (EBOV-GP-1F/EBOV-GP-1R), 57°C (primers by Trombley et al. [9]), or 50°C (nested PCR primers) for 30 s, 72°C for 30 s, and a 5-min final extension. PCR amplicons were purified with the DNA Clean & Concentrator-5 kit (Zymo Research) and visualized by 2% gel electrophoresis. Amplicons were cloned using the TOPO TA Cloning kit (Thermo Fisher Scientific), and Sanger sequencing of the cloned inserts was performed by Elim Biopharmaceuticals, Inc. (Hayward, CA). The primer sequences for the confirmatory RT-PCR assays and expected amplicon sizes are given in Table S1.

### Metagenomic next-generation sequencing.

For each whole-blood sample, 10 μl of resuspended extract was treated with 1 unit of Turbo DNase (Ambion) at 37°C for 30 min and inactivated with 1.1 μl of DNase inactivation reagent for 5 min. RNA was reverse transcribed with SuperScript III reverse transcriptase (Life Technologies) using a random primer attached to a linker adapter (Sol-PrimerA, 5′-GTTTCCCACTGGAGGATA-N9-3′), followed by second-strand DNA synthesis with Sequenase DNA polymerase (Affymetrix), as previously described (10). Metagenomic next-generation sequencing (mNGS) libraries were constructed from amplified cDNA using the Nextera XT DNA library preparation kit (Illumina). Dual-indexed barcodes were employed to enable pooling of libraries and to assign reads to individual samples after sequencing. Multiplexed barcoded mNGS libraries were sequenced as 150-bp (bp) paired-end (PE) runs on a HiSeq 2500 instrument (Illumina), with up to 14 sample libraries multiplexed per lane.

### Capture probe enrichment of EBOV.

To enhance genome recovery, we enriched select mNGS libraries for EBOV sequences using XGen biotinylated lockdown capture probes (IDT Technologies), followed by Illumina MiSeq sequencing of the enriched libraries, with up to 10 enriched multiplexed sample libraries per lane. Clinical samples were chosen for enrichment if (i) at least 1 EBOV read was identified in the initial mNGS run by BLASTn alignment to a 2014 Boende outbreak viral reference genome (KP271018) at an E value cutoff of 1 × 10^−8^ and (ii) the sequenced EBOV reads yielded incomplete (<99%) viral genome coverage, as samples with ≥99% genome recovery did not require enrichment. The 223 capture probes were designed to tile across all fully sequenced EBOV genomes in the National Center for Biotechnology Information (NCBI) GenBank database as of 18 December 2014 (see Table S2). Enrichment was performed using the XGen lockdown protocol and SeqCap EZ Hybridization and Wash kit (Roche Molecular Systems) according to the manufacturer’s instructions with a 24-h incubation time for hybridization, followed by 150-bp PE sequencing on a MiSeq instrument (Illumina). A separate no-template control (NTC) sample consisting of extraction buffer was used to assess for cross-contamination.

### Metagenomic sequencing analysis.

Metagenomic sequencing data were analyzed for pathogens using the sequence-based ultrarapid pathogen identification (SURPI) bioinformatics pipeline (11). Both read 1 and paired-end read 2 were analyzed independently for purposes of SURPI analysis. A 75-bp segment from base positions 10 to 75 was used for nucleotide alignment, followed by recovery of the entire 150-bp read length for viral genome assembly. After preprocessing to exclude low-quality, low-complexity, and adapter sequences, human sequences were computationally subtracted from the mNGS data. This was followed by nucleotide alignment using an edit distance of 12 to identify reads from viruses, bacteria, fungi, or parasites. Microbial references in NCBI GenBank corresponding to false-positive alignments were filtered out by high-stringency BLAST alignment of candidate reads, one per unique GenBank identifier or accession number, at an E value of 1 × 10^−8^. Remaining reads were then taxonomically classified to the species, genus, or family level using the lowest common ancestor algorithm. Potentially novel viruses with divergent sequences were searched for by translated nucleotide alignment against known reference sequences present in the GenBank viral protein database (June 2013 build). No reads to EBOV, Plasmodium falciparum, or other blood-borne viral pathogens were detected in the 4 negative UCSF patient samples processed in parallel with the primary mNGS run nor were reads to EBOV detected in the NTC sample during the subsequent capture probe enrichment sequencing run.

### RT-PCR confirmation of Orungo virus.

Qualitative RT-PCR testing was performed to confirm the finding of Orungo virus in a patient sample (*n* = 1), using a newly designed primer set that targeted segment 1 of the virus (F, 5′-ATGGAACGGGAAAAGACGGG-3′; R [2,253 to 2,273 bp], 5′-CCGCGCGATGATTCTTCCTA-3′). The RT-PCR assay was performed using the Qiagen one-step RT-PCR kit (Qiagen) in a 25-μl total reaction volume and with 10 μM each primer, according to the manufacturer’s instructions. Conditions for the RT-PCR were as follows: 50°C for 30 min and 95°C for 15 min, followed by 40 cycles of 94°C for 15 s, 55°C for 30 s, and 72°C for 1 min, followed by a final incubation at 72°C for 10 min. PCR products were evaluated by 2% agarose gel electrophoresis. Bands of the expected size (311 bp) were purified prior to sequencing using the Invitrogen PureLink Quick Gel Extraction kit (Thermo Fisher Scientific) according to the manufacturer’s protocol. Purified PCR products were Sanger sequenced in both forward and reverse orientations using the same primer sequences as used for PCR at 2 nM concentration.

### Statistical analyses.

Two-sided Fisher’s exact test was used to compare clinical characteristics between probable or confirmed EVD and non-EVD cases and between EVD/malaria coinfected and singly infected EVD cases. A *P* value of <0.05 was used as the cutoff for statistical significance.

### Genome assembly and phylogenetic analysis.

Genome assembly was performed using the Geneious v10.2.2 software package (12) and a 2014 DRC Ebola strain reference (KP271018). We mapped all reads aligning to EBOV from each patient sample to generate individual consensus EBOV genomes, of which those with coverage of ≥50% were retained for phylogenetic analysis. All complete EBOV genome sequences as of August 2017 were downloaded from GenBank. As more than 1,000 genome sequences were available for the 2013 to 2016 West Africa epidemic, we selected 36 representative sequences comprising up to 5 sequences per year and per outbreak location (i.e., Guinea, Sierra Leone, or Liberia) for phylogenetic analysis. For each EBOV reference genome, the coding protein sequences were extracted and then concatenated (NP-VP35-VP40-GP-VP30-VP24-L) to yield complete coding genome sequences. These concatenated coding sequences were aligned together with the new EBOV coding sequences from the 2014 DRC outbreak generated here, using MAFFT version 5.0 (13). We inferred a maximum likelihood (ML) phylogeny from this alignment using RAxML version 8 (14) under a general time reversible nucleotide substitution model and a gamma-distributed model of among site rate variation (GTR+Γ), as determined by jModelTest2 (15, 16). Statistical support for nodes in the ML phylogeny was evaluated using a bootstrapping approach with 100 replicates.

Next, we evaluated the temporal molecular clock signal of the alignment using TempEst (17), which regresses the sample collection dates against the root-to-tip genetic distances from the ML phylogeny. The plot indicated that the data set contained a sufficient temporal signal for a molecular clock analysis. A molecular clock phylogeny was estimated from the alignment using the Bayesian Markov chain Monte Carlo (MCMC) approach implemented in BEAST v1.8.4 (18). We computed an MCMC for 100 million steps, with sampling of parameters and trees every 10,000 steps. For the MCMC analysis, we used the SRD06 nucleotide substitution model, an uncorrelated log-normal relaxed molecular clock model (with a noninformative continuous-time Markov chain reference prior placed on the molecular clock rate parameter), and a Bayesian SkyGrid coalescent tree prior. The program Tracer v1.6 was used to check MCMC convergence, and the program TreeAnnotator as implemented in BEAST v1.8.4 was used to compute a maximum clade credibility tree, after removal of 20% of the chain as burn-in.

### Accession number(s).

The 14 complete and partial EBOV genomes recovered in this study have been submitted to NCBI GenBank under accession numbers MK044558 to MK044571. The mNGS reads with human sequences removed have been submitted to the NCBI Sequence Read Archive (BioProject accession number PRJNA557303).

## RESULTS

### Clinical and epidemiological analysis.

From 13 August to 8 September 2014, 37 of 70 patients with suspected EVD were documented as either confirmed (*n* = 22) or probable (*n* = 15) EVD cases, of which 5 were male and 32 were female with an average age of 35.4 (± 16.1) years. Overall, 38 of 70 patients who presented during the outbreak died. Excluding the established non-EVD cases with negative EBOV qRT-PCR_DRC_ testing (*n* = 7, all survivors), this yielded an outbreak case fatality rate of 60.3% (38 of 63 confirmed, probable, or indeterminate EVD cases). Among the 37 cases defined as confirmed or probable EVD, 23 patients died, yielding a comparable fatality rate of 62.2% (23 of 37) when adjusted for EBOV-attributable cases. These 23 patients included 3 who were males and 20 who were female, including a child <1 year of age.

Among the 37 confirmed or probable EVD patients, reported clinical data were available for 35; 91.4% had fever, 34.3% headache, 68.6% diarrhea or vomiting, 37.1% abdominal pain, 48.6% fatigue, 37.1% myalgia, and 37.1% with at least one bleeding manifestation (see Table S3 in the supplemental material). EVD patients (*n* = 35) were more likely than non-EVD patients (*n* = 7) to present with symptoms of fatigue (*P* < 0.03) and to die from their acute illness (*P* < 0.001) (Table 1). No significant differences in clinical characteristics were found when comparing EBOV-malaria coinfected to EBOV singly infected cases (see Table S4). The majority of the 37 total probable and confirmed EVD cases were reported in local clinics from Lokolia (24 cases) or Watsi Kengo (6 cases). Four cases were from Boende town, and the remaining 3 cases were from other areas in the district.

**TABLE 1:** Cases of Ebola virus disease (probable or confirmed) according to reported signs and symptoms

Symptom	No. (%) of patients^*a*^			*P* value^*b*^		
	Non-EVD cases (*n* = 7)	Probable EVD cases (*n* = 13)	Confirmed EVD cases (*n* = 22)	Probable vs. non-EVD	Confirmed vs. non-EVD	Confirmed/probable vs. non-EVD
Fever	6 (86)	12 (92)	20 (91)	0.48	0.44	0.41
Headache	0 (0)	4 (31)	8 (36)	0.15	0.07	0.08
Diarrhea	2 (29)	11 (85)	13 (59)	**0.02**	0.13	0.05
Abdominal pain	0 (0)	5 (39)	8 (36)	0.08	0.07	0.06
Vomiting	2 (29)	11 (85)	12 (55)	**0.02**	0.18	0.07
Fatigue	0 (0)	8 (62)	9 (41)	**0.01**	0.05	**0.02**
Anorexia	0 (0)	6 (46)	4 (18)	**0.04**	0.31	0.12
Muscle pain	0 (0)	5 (39)	8 (36)	0.08	0.07	0.08
Dysphagia	0 (0)	5 (39)	5 (23)	0.08	0.22	0.12
Dyspnea	0 (0)	1 (8)	4 (18)	0.65	0.31	0.38
Cough	0 (0)	0 (0)	2 (9)	1.00	0.57	0.69
Skin rash	0 (0)	0 (0)	2 (9)	1.00	0.57	0.69
Bleeding from injection site	0 (0)	0 (0)	2 (9)	1.00	0.57	0.69
Gingival bleeding	0 (0)	0 (0)	2 (9)	1.00	0.57	0.69
Conjunctival bleeding	0 (0)	1 (8)	2 (9)	0.65	0.57	0.57
Melena	0 (0)	5 (39)	5 (23)	0.08	0.22	0.12
Hematemesis	0 (0)	2 (15)	4 (18)	0.41	0.31	0.31
Epistaxis	0 (0)	1 (8)	3 (14)	0.65	0.42	0.47
Vaginal bleeding	0 (0)	0 (0)	2 (9)	1.00	0.57	0.69
Other types of bleeding	0 (0)	0 (0)	2 (9)	1.00	0.57	0.69
Deceased	0 (0)	8 (62)	15 (68)	**0.01**	**2.20E−03**	**1.87E−03**

a From the 70 patients in the study, 65 had clinical information to perform statistical testing.

b *P* values using Fisher’s exact test were calculated using patients with probable or confirmed EVD cases compared to negative EVD patients. Significant symptom severity measured at a *P* value of <0.05 is marked in boldface font.

### Metagenomic next-generation sequencing of EBOV samples.

Among the 70 patients in the study, 31 were initially tested for EBOV in the Democratic Republic of the Congo by qRT-PCR_DRC_ testing from whole-blood samples, of which 20 (64.5%) were positive (Table 2). RNA extracts from all 70 patients were then shipped to the United States at room temperature in a Biomatrica RNAstable matrix for mNGS testing, but were not fully dried prior to shipment as recommended by the manufacturer. Partial RNA degradation occurred during shipment to the United States, as analysis of RNA integrity numbers (RIN) for 8 of 12 available sample extracts, selected due to discrepant qRT-PCR results between DRC and U.S. assays (i.e., 12 EBOV qRT-PCR_DRC_-positive/qRT-PCR_US_-negative samples), revealed evidence of RNA degradation in all 8 (100%) (see Table S5). Thus, EBOV qRT-PCR_US_ of the shipped RNA extracts yielded positive results for only 7 of 20 (35.0%) previously positive samples but also identified an additional 14 positive samples that had either tested negative (*n* = 3) or not been tested (*n* = 11) in the Democratic Republic of the Congo. In total, 34 samples (48.6%) were EBOV positive by qRT-PCR_DRC_ and/or qRT-PCR_US_ testing, but only 21 samples were positive by qRT-PCR_US_ testing alone.

**TABLE 2:** RT-PCR results, mNGS reads, and viral genome alignment coverage from EBOV testing for 70 patient samples from the outbreak

Sample	mNGS			mNGS with probe enrichment					PCR results					mNGS result for ZEBOV^*b*^	Final classification^*c*^	Clinical outcome
	Total no. of reads	No. of reads to ZEBOV	% coverage to ZEBOV	Total # of reads	No. of reads to ZEBOV	Fold enrichment	% coverage to ZEBOV	% increase in coverage	qRT-PCR (DRC)	qRT-PCR (US)	Confirmatory RT-PCR (US)	*C_T_*^*a*^	Calculated viral load			
BOE_007	13,530,534	10	4.2	149,261	1,459	146	51.3	47.1	+	+	+	36.2	6.25E+04	+	Confirmed EVD	Deceased
BOE_011	21,029,450	184,925	99.8						NT^*d*^	+	+	26.4	3.31E+07	+	Confirmed EVD	Deceased
BOE_013	13,217,428	2,058	52.2	8,906,945	302,816	147	84.4	32.2	+	+	+	33.3	4.00E+05	+	Confirmed EVD	Alive
BOE_015	14,040,176	530	11.5	314,033	114,990	217	62.4	50.9	NT	+	–	34	2.55E+05	+	Confirmed EVD	Alive
BOE_016	11,644,602	5	1.4	87,398	860	172	40.6	39.3	+	–				+	Confirmed EVD	Deceased
BOE_017	19,519,018	4,181	54.2	262,011	207,168	50	76.2	22.0	NT	+	–	32	9.18E+05	+	Confirmed EVD	Deceased
BOE_021	14,305,256	2	1.1	49,814	1,298	649	49.7	48.7	NT	+	–	36.3	5.86E+04	+	Confirmed EVD	Deceased
BOE_023	22,032,448	14,057	100.0						NT	+	+	25.3	6.69E+07	+	Confirmed EVD	Deceased
BOE_034	8,537,192	2	0.6	20,573	1,574	787	49.8	49.2	+	+	–	38	1.97E+04	+	Confirmed EVD	Alive
BOE_035	13,874,416	1	0.4	203,976	97	97	17.2	16.8	+	–				+	Confirmed EVD	Deceased
BOE_036	18,921,752	286,723	100.0						NT	+	+	23.4	2.26E+08	+	Confirmed EVD	Deceased
BOE_037	9,992,378	1	0.4	97,227	52	52	10.7	10.3	NT	+	+	35.7	8.60E+04	+	Confirmed EVD	Deceased
BOE_039	9,190,338	1	0.4	107,133	85	85	18.3	17.9	+	+	NT	36.2	6.25E+04	+	Confirmed EVD	Deceased
BOE_045	16,321,890	0							+	–				–	Confirmed EVD	Deceased
BOE_046	13,683,244	0							+	–				–	Confirmed EVD	Deceased
BOE_063	17,469,266	0							+	+	NT	35.1	1.26E+05	–	Confirmed EVD	Alive
BOE_064	15,368,202	1	0.4	100,420	80		16.1	15.7	–	+	NT	40.9	3.08E+03	+	Confirmed EVD	Alive
BOE_065	19,320,118	20	7.7	140,155	2,685		27.6	19.9	–	+	+	29.1	5.88E+06	+	Confirmed EVD	Alive
BOE_069	26,924,516	855	5.6	80,760	29,174		12.2	6.6	+	+	+	33.6	3.30E+05	+	Confirmed EVD	Deceased
BOE_070	19,749,830	73,147	95.5	1,494,384	1,466,174		97.9	2.4	+	+	+	24.4	1.19E+08	+	Confirmed EVD	Deceased
BOE_078	26,936,926	1	0.4	5,817	863		63.4	63.0	–	+	+	36.5	5.15E+04	+	Confirmed EVD	Alive
BOE_084	18,760,962	419	39.1	35,536	9,805		84.3	45.2	NT	+	+	27.6	1.53E+07	+	Confirmed EVD	Unknown
BOE_006	13,861,466	0							+	–				–	Probable EVD	Alive
BOE_012	17,121,118	0							+	–				–	Probable EVD	Alive
BOE_020	13,128,106	0							+	–				–	Probable EVD	Deceased
BOE_030	17,942,078	0							NT	+	NT	38.1	1.85E+04	–	Probable EVD	Deceased
BOE_033	16,194,794	0							+	–				–	Probable EVD	Alive
BOE_048	18,787,656	5	1.2	68,449	211	42	19.5	18.3	NT	–				+	Probable EVD	Deceased
BOE_053	12,228,468	0							NT	+	NT	35.4	1.04E+05	–	Probable EVD	Alive
BOE_055	19,528,904	0							NT	+	NT	34.8	1.53E+05	–	Probable EVD	Deceased
BOE_060	19,274,440	0							+	–				–	Probable EVD	Deceased
BOE_061	17,877,356	0							+	–				–	Probable EVD	Deceased
BOE_062	18,406,940	0							+	–				–	Probable EVD	Alive
BOE_067	20,992,354	0							+	–				–	Probable EVD	Deceased
BOE_068	18,685,602	0							+	–				–	Probable EVD	Deceased
BOE_073	20,308,534	1	0.4	34,880	2,044		72.8	72.4	NT	–				+	Probable EVD	Unknown
BOE_079	24,925,148	1	0.4	11,030	2,674		75.3	74.9	–	–				+	Probable EVD	Deceased
BOE_001	22,533,096	0							NT	–				–	Indeterminate EVD	Deceased
BOE_005	24,918,444	0							NT	–				–	Indeterminate EVD	Deceased
BOE_008	21,723,630	0							NT	–				–	Indeterminate EVD	Deceased
BOE_009	18,807,290	0							NT	–				–	Indeterminate EVD	Deceased
BOE_010	15,523,496	0							NT	–				–	Indeterminate EVD	Deceased
BOE_014	24,153,028	0							NT	–				–	Indeterminate EVD	Deceased
BOE_026	30,814,980	0							NT	–				–	Indeterminate EVD	Alive
BOE_027	24,490,500	0							NT	–				–	Indeterminate EVD	Alive
BOE_029	15,545,630	0							NT	–				–	Indeterminate EVD	Alive
BOE_038	13,575,740	0							NT	–				–	Indeterminate EVD	Deceased
BOE_040	10,043,588	0							NT	–				–	Indeterminate EVD	Deceased
BOE_041	14,152,364	0							NT	–				–	Indeterminate EVD	Deceased
BOE_042	14,562,248	0							NT	–				–	Indeterminate EVD	Deceased
BOE_043	14,959,330	0							NT	–				–	Indeterminate EVD	Alive
BOE_044	21,893,586	0							NT	–				–	Indeterminate EVD	Deceased
BOE_049	15,698,664	0							NT	–				–	Indeterminate EVD	Deceased
BOE_050	19,856,236	0							NT	–				–	Indeterminate EVD	Deceased
BOE_051	19,560,668	0							NT	–				–	Indeterminate EVD	Alive
BOE_052	13,683,924	0							NT	–				–	Indeterminate EVD	Alive
BOE_054	5,551,494	0							NT	–				–	Indeterminate EVD	Deceased
BOE_056	16,011,850	0							NT	–				–	Indeterminate EVD	Alive
BOE_057	18,324,516	0							NT	–				–	Indeterminate EVD	Alive
BOE_058	17,418,264	0							NT	–				–	Indeterminate EVD	Deceased
BOE_059	18,728,830	0							NT	–				–	Indeterminate EVD	Unknown
BOE_086	18,849,366	0							NT	–				–	Indeterminate EVD	Unknown
BOE_087	18,183,444	0							NT	–				–	Indeterminate EVD	Unknown
BOE_022	17,245,420	0							–	–				–	Non-EVD	Alive
BOE_028	20,692,650	0							–	–				–	Non-EVD	Alive
BOE_066	15,492,274	0							–	–				–	Non-EVD	Alive
BOE_074	18,121,652	0							–	–				–	Non-EVD	Alive
BOE_075	6,756,328	0							–	–				–	Non-EVD	Alive
BOE_076	11,615,728	0							–	–				–	Non-EVD	Alive
BOE_077	15,569,022	0							–	–				–	Non-EVD	Alive

a *C_T_*, cycle threshold.

b ZEBOV, Zaire Ebola virus.

c Cases were classified as “confirmed EVD” if positive by at least 2 of the 3 following molecular tests: mNGS, qRT-PCR_DRC_, and qRT-PCR_US_; “probable EVD” if positive by 1 of the 3 tests; “non-EVD” if negative by all 3 tests (and qRT-PCR_DRC_ testing had been performed); or “indeterminate EVD” otherwise.

d NT, not tested.

We attempted to confirm the qRT-PCR_US_ results (21 of 70 positive) with a series of follow-up RT-PCRs and Sanger sequencing of amplicons of the expected size visualized by gel electrophoresis (see Fig. S2A). First, 56 available samples out of 70 were independently screened for EBOV positivity using a *de novo* designed primer set directed against the glycoprotein gene (EBOV-GP-1F/EBOV-GP-1R). Five samples tested positive and were confirmed as EBOV by Sanger sequencing, all 5 of which had previously tested qRT-PCR_US_ positive. Among the 16 remaining positive qRT-PCR_US_ samples, 11 had a sufficient amount of RNA remaining for repeat RT-PCR testing using the primers designed by Trombley, et al. (9); 10 of the 11 were tested by repeat RT-PCR, of which an additional 6 samples were found to be positive and confirmed as EBOV by Sanger sequencing (Fig. S2B). Finally, we tested the available remaining RNA from 3 low-titer samples also with only 1 or 2 mNGS reads (BOE_021, BOE_034, and BOE_037) using nested PCR with primers designed from these few mNGS reads (Fig. S2C). Among these 3 samples, we recovered one additional positive (BOE_037), subsequently confirmed as EBOV by Sanger sequencing. In summary, from 16 of 21 initial qRT-PCR_US_ samples with sufficient RNA remaining, we confirmed 12 of the 16 as positive for EBOV by repeat RT-PCR and Sanger sequencing.

An average of 17,267,003 (±4,727,295 standard deviation [SD]) raw mNGS reads were generated per whole-blood sample, with at least one EBOV read identified in 22 of 70 samples (31.4%) (Table 2). Although the RNA was degraded, we only kept preprocessed reads with an average quality score of 30 or higher for the downstream pathogen identification and viral genome assembly steps. The number of recovered EBOV reads per sample was on average 8,099 (±41,246 SD), with a range of 1 to 286,723. The proportions of qRT-PCR_US_-positive and mNGS-positive samples following partial RNA degradation during shipment were similar overall (21 of 70 [30.0%] versus 22 of 70 [31.4%], respectively), with a concordance of 87.1%. To enhance viral genome recovery, subsequent enrichment using EBOV-specific probes was performed on 19 of the 22 (86.4%) samples containing EBOV reads that had yielded incomplete (<99%) viral genome coverage; the remaining 3 EBOV samples had >99% coverage from mNGS alone and so did not need additional enrichment. On average, probe enrichment increased EBOV coverage by 34.3%, yielding an additional 11 EBOV genomes, 6 with coverage of ≥50%.

Reads corresponding to P. falciparum were detected in 21 of 70 patient samples (30.0%), with an average of 4,548 (±29,980 SD) and range of 1 to 248,696 reads per sample (see Table S6 and Fig. S3). Additional viral reads detected in the mNGS data corresponded to human pegivirus 1 (HPgV1) (*n* = 10, 14.3%), hepatitis B virus (HBV) (*n* = 2, 2.9%), and Epstein-Barr virus (EBV) (*n* = 9, 12.9%), In total, 15 of 37 (40.5%) patients with confirmed or probable EBOV infection had additional reads from infectious agents, of which 9 of 37 (24.3%) were coinfections with P. falciparum.

One EBOV qRT-PCR_US_-negative sample had identifiable mNGS reads for Orungo virus, a rarely reported orbivirus in the *Reoviridae* family (Table S6 and Fig. S4). Confirmatory PCR and Sanger sequencing of the resulting amplicon confirmed the presence of Orungo virus in the patient sample. Although qRT-PCR_US_ negative, EBOV reads were detected in the Orungo virus sample by mNGS.

### Genome assembly and phylogenetic analysis of EBOV.

We aligned the concatenated coding genome sequences of the 14 newly assembled whole and partial EBOV genome sequences generated in this study with 71 publicly available EBOV genomes, including 5 previously published sequences from the 2014 outbreak and a curated set of 36 representative sequences from the 2013 to 2016 West Africa epidemic. The maximum likelihood phylogeny consisted of many well-supported nodes and exhibited a general topology that agreed with previous studies (see Fig. S5) (19, 20). All of the 2014 Boende outbreak sequences formed a monophyletic clade that was most closely related to EBOV strains isolated in Gabon and the Democratic Republic of the Congo in 1994 to 1996 with branch bootstrap supports of 100% (Fig. S5).

**FIG 1:**
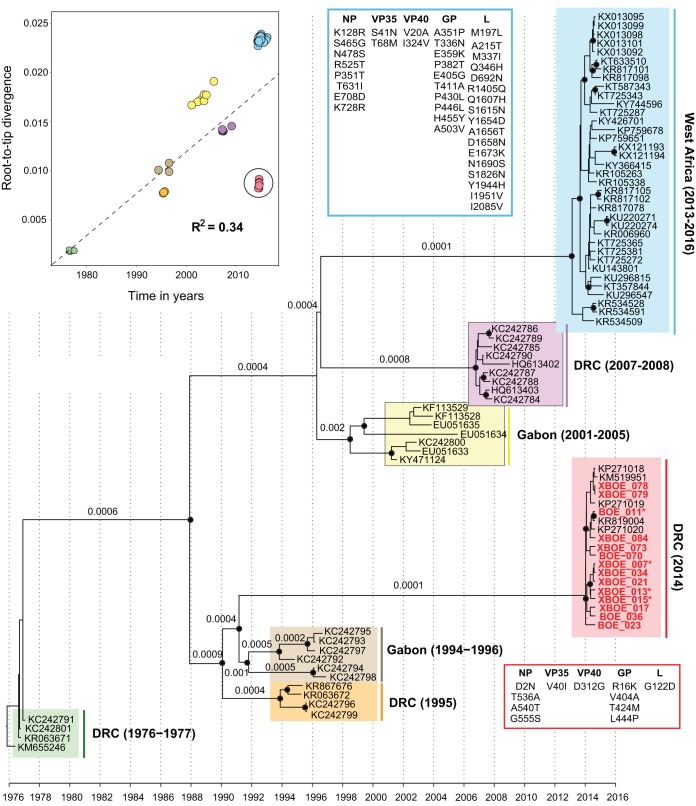
Temporal signal and molecular clock phylogeny of the Zaire ebolavirus lineage. (Top left) Regression of sample collection dates against root-to-tip genetic distances obtained from an estimated maximum likelihood phylogeny. The phylogeny shown here was estimated using a Bayesian molecular clock approach from the complete coding genome sequences of 85 Zaire ebolaviruses, including 18 sequences collected from the 2014 outbreak in the Democratic Republic of the Congo. Black circles at phylogenetic nodes indicate Bayesian posterior probabilities of >0.75, and numbers below or above phylogenetic branches indicate branch-specific evolutionary rates estimated from the relaxed molecular clock model. Genome sequences generated in this study are in bold.

A regression analysis of genetic divergence versus sequence sampling dates revealed that the branch immediately ancestral to the 2014 DRC sequences was shorter than expected, with genetic distances from the root comparable to those of viruses sampled in the 1990s. Consequently, the 2014 Boende EBOV sequences fell below the regression line (Fig. 1), implying a markedly lower rate of molecular evolution on the branch leading to the 2014 outbreak. The estimated molecular clock tree (Fig. 1) was also well supported and exhibited a tree topology similar to that of the ML phylogeny (Fig. S5) and previous studies (19, 20). The estimated mean rate of molecular evolution across all branches in the phylogeny was 4.7 × 10^−4^ substitutions per nucleotide site per year (95% highest posterior density [HPD] interval = 3.4 × 10^−4^ to 5.7 × 10^−4^). The evolutionary rate estimated for the long branch leading to the 2014 Boende outbreak was approximately four times lower (at 1 × 10^−4^ substitutions/site/year, 95% HPD interval = 8 × 10^−5^ to 1.6 × 10^−4^) than the mean branch rate. In contrast, the evolutionary rate estimated for the long branch ancestral to the 2013 to 2016 West Africa epidemic was 1 × 10^−3^ substitutions/site/year (95% HPD interval = 5.6 × 10^−4^ to 1.4 × 10^−3^). These estimated branch rates most likely represent EBOV evolution in one or more animal reservoir species and are distinct from the evolutionary rates estimated for individual lineages in human outbreaks (see reference 19 for more detailed discussion of this issue). Unlike the West Africa epidemic, which was unusually long lived, it was not possible to reliably estimate an evolutionary rate specific to the 2014 Boende outbreak because of the limited timescale over which samples were obtained.

## DISCUSSION

A metagenomic sequencing approach is attractive for outbreak surveillance, given that all infectious pathogens are simultaneously detected on the basis of uniquely identifying RNA and/or DNA sequences. Previous studies using multiple different sequencing platforms have shown the ability to detect EBOV reads from whole-blood or plasma samples by mNGS (6, 21, 22). Here, we demonstrate that mNGS analyses of field-collected samples can be used to (i) recover 9 genomes from the 2014 Boende outbreak exceeding 50% coverage (the minimum threshold proposed as the standard for a sequenced draft viral genome [23]), (ii) detect EBOV with high sequencing depth (17.3 ± 4.7 SD million reads) with comparable sensitivity to PCR, and (iii) identify coinfections from both well-recognized (P. falciparum) and novel/uncommon (e.g., Orungo virus) pathogens. Our results also indicate that useful sequencing data can still be extracted from RNA samples collected in the field, despite partial degradation from inadequate handling, storage, and/or loss of cold chain (24).

The overall topology of the EBOV phylogeny and relative placement of the 2014 Boende lineage characterized here is consistent with prior reports (6, 19, 20). Analysis of a larger data set consisting of 9 viral genomes strengthens a previously described finding (based on 4 genomes [20]) of a markedly lower evolutionary rate for the 2014 Boende strain, or more precisely, for the phylogenetic branch immediately basal to the 2014 outbreak clade. Here, we used a relaxed molecular clock approach to quantify this rate and found it to be on average ∼5 to ∼10 times lower than the rate estimated for other long internal branches in the EBOV phylogeny, such as those immediately ancestral to the 2013 to 2016 West Africa, 2007 to 2008 DRC, and 2001 to 2005 Gabon outbreak clades (Fig. 1). Thus, the lineage that gave rise to the 2014 Boende outbreak appears to exhibit different molecular evolutionary dynamics than other EBOV lineages. Little is known about the maintenance of Ebola viruses in nonhuman reservoir species, although a novel Ebola virus was recently discovered in a fruit bat (25). It is possible that Ebola virus circulation among one or more animal reservoir species will result in long viral generation times or altered selective pressures compared to those for direct transmission among humans. An alternative hypothesis is that the 2014 Boende strain has an intrinsically lower rate of spontaneous mutation (20). Current molecular sequence data alone cannot discriminate between these two nonmutually exclusive hypotheses; thus, further comparative experimental studies of these EBOV strains *in vitro* are likely required.

Following shipment to the United States, similar numbers of samples were found to be EBOV positive by qRT-PCR_US_ (21 of 70 [30.0%]) and mNGS (22 of 70 [31.4%]), with high (87.1%) concordance. We were able to confirm 11 of the 15 qRT-PCR_US_-positive samples with remaining RNA available for repeat RT-PCR and Sanger sequencing. The remaining 4 were not positive on confirmatory RT-PCR testing, likely due to sample degradation from multiple rounds of aliquoting and freeze-thaw cycles. The comparable sensitivity of mNGS relative to single-target PCR at relatively high sequencing depths (an average of 17.3 ± 4.7 SD million reads for the present study) was demonstrated previously (26–28), albeit not with field-collected partially degraded samples. In addition, among the 63 cases examined with suspected Ebola hemorrhagic fever, 26 were negative by both mNGS and PCR testing in the United States (and had not been tested on site in the Democratic Republic of the Congo) and were thus classified as “indeterminate EVD” (Table 2). The failure to detect EBOV in these patients is most likely due to sample degradation during shipment to the United States, although low EBOV copy number remains another possible explanation.

Using mNGS, multiple infectious agents other than Ebola were detected in patient samples with suspected viral hemorrhagic fever. Among the infectious agents detected, only P. falciparum infection (malaria) is an established cause of hemorrhagic fever with symptoms that can overlap those of EVD. In total, 15 coinfections and 9 standalone infections with P. falciparum out of 70 with suspected EVD were identified. Previous studies of the impact of coinfection with EBOV and P. falciparum have been conflicting. In one study, the concurrent presence of malaria in EVD patients had a higher mortality rate than standalone infections by either malaria or EBOV (29). This contrasts with findings from another study in which EVD patients with the highest levels of P. falciparum parasitemia had the highest survival rate. In this study, we observed no significant differences in disease severity or mortality rates between malaria/EVD coinfected and singly infected EVD patients. There may have been insufficient statistical power to detect an association given the relatively small sample size of the 2014 Boende cohort compared to the West Africa epidemic cohorts studied in the aforementioned reports. Alternatively, the patients in the study may have been treated recently or concurrently for malaria, although these data were not available. The detection of other coinfections from HBV (30), EBV (31), and HPgV1 (32) is likely incidental to the acute illness in the 70 EVD-suspected cases in our cohort.

Orungo virus is a mosquito-borne arbovirus that is known to infect humans, as antibodies to the virus have been reported in human samples (33). Isolated case reports of acute febrile illness and neurological disease (34), but not hemorrhagic fever, were also previously described in association with Orungo virus infection. In the present study, the whole-blood sample positive for Orungo virus was collected from a patient presenting with an acute febrile illness who subsequently died from massive hemorrhage and dehydration. The Orungo virus sample was negative for EVD by initial RT-PCR screening done in the Democratic Republic of the Congo and the United States; however, probe-enriched mNGS testing yielded positive results for EBOV. Unfortunately, as samples were collected primarily for diagnostic purposes, repeat blood samples, including for the patient with Orungo virus infection, were not available. We believe that mNGS cross-contamination is unlikely to explain these discrepant results, as >75% of the viral genome was ultimately recovered by probe enrichment and phylogenetic analysis positioned the EBOV strain on a unique branch. This suggests that EVD may indeed be the proximate cause of the patient’s death, although we cannot rule out an additive effect from concurrent Orungo virus infection.

In summary, mNGS testing for investigating viral outbreaks such as EBOV casts a broad net for detection of potential pathogens and thus may be particularly useful given that a large proportion of suspected patients during a viral outbreak may in fact be infected with a different pathogen. Even at the height of the West Africa epidemic (October 2014 to March 2015), 23% of patients in Liberia were diagnosed with laboratory-confirmed *Plasmodium* infection (malaria) alone and not EVD, similar to the percentage of patients with documented PCR-positive EBOV infection (24.5%) (35). Identification of infections other than EBOV and/or coinfections using mNGS can facilitate more timely differential diagnosis and early triaging of patients in an outbreak setting. Indeed, findings suggest that EBOV in West Africa negatively affected the treatment of malaria cases as a result of reduced health care capacity (36), likely increasing the morbidity caused by the 2014 to 2016 epidemic. The utility of mNGS analyses in the field may likely lie in early investigation of unknown outbreaks, in which only a few cases may need to be examined in order to identify the etiologic agent. Genome recovery of the outbreak virus facilitates tracking of evolution and spread, as demonstrated here and in other studies (37, 38). Our results suggest that mNGS can serve as a front-line surveillance tool for informing clinical and public health responses to disease outbreaks such as that caused by the 2014 Boende EBOV strain.

## Supplementary Material

Supplemental file 1

Supplemental file 2

Supplemental file 3
