# Supplementary material for: Metagenomic Next-Generation Sequencing of the 2014 Ebola Virus Disease Outbreak in the Democratic Republic of the Congo
Source: J Clin Microbiol. 2019 Aug 26;57(9):e00827-19. doi: 10.1128/JCM.00827-19 (PMC6711896; doi:10.1128/JCM.00827-19)
Supplement: Supplemental file 1 [file JCM.00827-19-s0001.pdf]

SUPPLEMENTARY MATERIAL

Metagenomic Next-Generation Sequencing of the 2014 Ebola Virus Disease Outbreak in the Democratic Republic of the Congo

Tony Li[1,2†], Placide Mbala-Kingebeni[3†], Samia N. Naccache[1,2], Julien Thézé[4], Jerome Bouquet[1,2], Scot Federman[1,2], Sneha Somasekar[1,2], Guixia Yu[1,2] Claudia Sanchez-San Martin[1,2], Asmeeta Achari[1,2], Bradley S. Schneider[5], Anne W. Rimoin[6], Andrew Rambaut[7], Justus Nsio[8], Prime Mulembakani[3], Steve Ahuka-Mundeke[3], Jimmy Kapetshi[3], Oliver G. Pybus[4], Jean-Jacques Muyembe-Tamfum[3], and Charles Chiu[1,2,8*]

[1]Department of Laboratory Medicine, University of California, San Francisco, CA 94107, USA

[2]UCSF-Abbott Viral Diagnostics and Discovery Center, San Francisco, CA 91407, USA

[3]Institut National de Recherche Biomédicale, Kinshasa, Democratic Republic of the Congo

[4]Department of Zoology, University of Oxford, Oxford, UK

[5]Etiologic, Inc, San Francisco, CA 94104, USA

[6]Department of Epidemiology, School of Public Health, University of California, Los Angeles, CA 90095

[7]Institute of Evolutionary Biology, University of Edinburgh, King's Buildings, Edinburgh, EH9 3FL, UK

[8]Ministry of Public Health, Kinshasa, Democratic Republic of the Congo

[8]Department of Medicine, Division of Infectious Diseases, University of California, San Francisco, CA 94107, USA

[†]these authors contributed equally to the manuscript

Table S1. EBOV PCR primers used for confirmatory RT-PCR and Sanger sequencing.

EBOV-GP-1F	TGG GCT GAA AAC TGC TAC AAT C
EBOV-GP-1R	TCC CGT TCC TGA TAC TTT GTG
(expected amplicon size is 150 bp)	
F565	TCTGACATGGATTACCACAAGATC
R640	GGATGACTCTTTGCCGAACAATC
(expected amplicon size is 75 bp; Trombley (2010), et al.)	
BOE_034_F7186	CAACCCAAGCTGAACAACATC
BOE_034_F7207	ACC GCA GAA CAG ACA ACG AC
BOE_034_R7299	TGC TCG TGT TGG TGT TCT CTG
BOE_034_R7309	TCA GTG CCC TTG CTC GTG TT
(expected amplicon size is 123 bp for the outer primer set, 92 for the inner primer set)	
BOE_021_F7138	ACA ACA GCA CCC ACA ATA CAC
BOE_021_F7169	ACTTGACATCTCTGAGGCAAC
BOE_021_R7244	AGT GTC GGA GGT TGT GCT GT
(expected amplicon size is 106 bp for the outer primer set, 75 bp for the inner heminested primer set)	
BOE_037_R2134	TCC AGT GGG TCT GCC TCT T
BOE_037_F2021	AGC AAT CCA CCA CAC CAG TG
BOE_037_F2042	TCC ACT CAC GGA CAA TGA CAG
BOE_037_R2119	TCT TCG TTG ATT GGT GTC AGC
(expected amplicon size is 113 bp for the outer primer set, 77 bp for the inner primer set)	

Table S2. XGen biotinylated lockdown capture probe sequences by IDT Technologies for ZEBOV genome enrichment. Sequences were designed to tile across complete ZEBOV genomes in the National Center of Biotechnology Information (NCBI) Genbank database as of December 18,2014.

“Table S2 in a separate supplemental Excel .XLSX file”

Table S3. Clinical manifestations and laboratory testing for EBOV in 70 suspected EVD patients during the 2014 Boende outbreak.

“Table S3 in a separate supplemental Excel .XLSX file”

Table S4. Cases of Ebola virus disease (probable or confirmed) according to reported signs and symptoms. From the 70 patients in the study, 65 out of 70 had clinical information to perform statistical testing. P-values using Fisher's Exact Test were calculated between EVD/malaria co-infected patients and singly infected EVD patients (among patients with either probable or confirmed EVD). *, confirmed or probable EVD cases.

Symptoms	EVD* only (n=18)	Malaria/EVD* co-infection (n=7)	P-value (EVD only vs. malaria/EVD co-infection)
		no. patients [%]	
Fever	16 [88.9%]	6 [85.7%]	0.47
Headache	8 [44.4%]	1 [14.3%]	0.15
Diarrhea	12 [66.7%]	5 [71.4%]	0.36
Abdominal pain	7 [38.9%]	2 [28.6%]	0.33
Vomiting	13 [72.2%]	4 [57.1%]	0.28
Fatigue	9 [50.0%]	2 [28.6%]	0.23
Anorexia	3 [16.7%]	2 [28.6%]	0.32
Muscle pain	6 [33.3%]	2 [28.6%]	0.36
Dysphagia	4 [22.2%]	2 [28.6%]	0.36
Dyspnea	3 [16.7%]	1 [14.3%]	0.45
Cough	0 [0.0%]	1 [14.3%]	0.28
Skin rash	0 [0.0%]	1 [14.3%]	0.28
Bleeding from injection site	0 [0.0%]	1 [14.3%]	0.28
Gingival Bleeding	0 [0.0%]	1 [14.3%]	0.28
Conjonctival bleeding	1 [5.6%]	1 [14.3%]	0.42
Melena	3 [16.7%]	2 [28.6%]	0.32
Haematemesis	3 [16.7%]	1 [14.3%]	0.45
Epistaxis	2 [11.1%]	1 [14.3%]	0.47
Vaginal Bleeding	1 [5.6%]	1 [14.3%]	0.42
Other types of bleeding	1 [5.6%]	0 [0.0%]	0.72
Deceased	10 [55.6%]	5 [71.4%]	0.28

Table S5. RNA Integrity Number (RIN) results from Agilent RNA 6000 Pico kit. Eight sample samples were chosen from 12 discrepant qRT-PCR results from testing in the DRC and US; insufficient RNA was available for RIN analysis of the remaining 4 samples. Agilent's definition for partial degradation is associated with a RIN of <5; strong degradation is associated with a RIN of <2. Not detectable (ND) were a result of the inability to detect measurable rRNA in the samples due to likely RNA degradation.

	**PCR Results**		**Agilent RNA Pico Results**	
Sample	qRT-PCR(DRC)	qRT-PCR (US)	RNA Integrity Number (RIN)	Definition
BOE_006	+	–	2.1	Strongly degraded
BOE_012	+	–	ND	Likely degraded
BOE_033	+	–	ND	Likely degraded
BOE_046	+	–	5.2	Partially degraded
BOE_060	+	–	2.5	Strongly degraded
BOE_061	+	–	ND	Likely degraded
BOE_067	+	–	ND	Likely degraded
BOE_068	+	–	ND	Likely degraded

Table S6. mNGS yield and reads from EBOV and other microorganisms in 70 patient samples.

	mNGS			Additional Viral Reads					
Sample	Total # of reads	# reads to ZEBOV	% Coverage to ZEBOV	GB virus C	Hepatitis B virus	Human herpesvirus 4 (EBV)	Orungo virus	*Plasmodium falciparum*	*Plasmodium falciparum* mNGS result
BOE_001	22,533,096	0	–	0	0	0	0	0	–
BOE_005	24,918,444	0	–	14,337	0	0	0	65	+
BOE_006	13,861,466	0	–	0	0	0	0	0	–
BOE_007	13,530,534	10	4.2%	0	0	0	0	0	–
BOE_008	21,723,630	0	–	0	0	0	0	0	–
BOE_009	18,807,290	0	–	0	0	0	0	0	–
BOE_010	15,523,496	0	–	0	0	0	0	1	+
BOE_011	21,029,450	184,925	99.8%	0	0	0	0	2	+
BOE_012	17,121,118	0	–	3	0	0	0	0	–
BOE_013	13,217,428	2,058	52.2%	0	0	0	0	0	–
BOE_014	24,153,028	0	–	0	0	0	0	0	–
BOE_015	14,040,176	530	11.5%	0	0	0	0	0	–
BOE_016	11,644,602	5	1.4%	1,627	0	0	0	0	–
BOE_017	19,519,018	4,181	54.2%	0	0	8	0	0	–
BOE_020	13,128,106	0	–	9,154	0	0	0	0	–
BOE_021	14,305,256	2	1.1%	0	0	0	0	0	–
BOE_022	17,245,420	0	–	0	0	0	0	2	+
BOE_023	22,032,448	14,057	100.0%	0	0	0	0	0	–
BOE_026	30,814,980	0	–	0	0	0	0	0	–
BOE_027	24,490,500	0	–	0	0	0	0	0	–
BOE_028	20,692,650	0	–	0	0	0	0	0	–
BOE_029	15,545,630	0	–	0	0	0	0	0	–
BOE_030	17,942,078	0	–	28,091	0	0	0	28,246	+
BOE_033	16,194,794	0	–	0	0	0	0	0	–
BOE_034	8,537,192	2	0.6%	0	0	30	0	0	–
BOE_035	13,874,416	1	0.4%	15,747	16,304	0	0	248,696	+
BOE_036	18,921,752	286,723	100.0%	0	0	0	0	0	–
BOE_037	9,992,378	1	0.4%	0	0	0	0	0	–
BOE_038	13,575,740	0	–	0	0	0	0	5	+
BOE_039	9,190,338	1	0.4%	0	0	0	0	0	–
BOE_040	10,043,588	0	–	0	0	0	0	8	+

	mNGS			Additional Viral Reads					
Sample	Total # of reads	# reads to ZEBOV	% Coverage to ZEBOV	GB virus C	Hepatitis B virus	Human herpesvirus 4 (EBV)	Orungo virus	*Plasmodium falciparum*	*Plasmodium falciparum* mNGS result
BOE_041	14,152,364	0	–	0	1,052	0	0	1	+
BOE_042	14,562,248	0	–	1	0	0	0	37	+
BOE_043	14,959,330	0	–	0	0	6	0	0	–
BOE_044	21,893,586	0	–	0	0	0	0	0	–
BOE_045	16,321,890	0	–	0	0	0	0	0	–
BOE_046	13,683,244	0	–	18,923	0	0	0	8	+
BOE_048	18,787,656	5	1.2%	0	0	12	0	0	–
BOE_049	15,698,664	0	–	0	0	0	0	0	–
BOE_050	19,856,236	0	–	0	0	0	0	0	–
BOE_051	19,560,668	0	–	0	0	0	0	0	–
BOE_052	13,683,924	0	–	0	0	0	0	1,163	+
BOE_053	12,228,468	0	–	0	0	0	0	1	+
BOE_054	5,551,494	0	–	0	0	66	0	0	–
BOE_055	19,528,904	0	–	0	0	0	0	0	–
BOE_056	16,011,850	0	–	0	0	0	0	117	+
BOE_057	18,324,516	0	–	0	0	0	0	24,713	+
BOE_058	17,418,264	0	–	0	0	2	0	0	–
BOE_059	18,728,830	0	–	0	0	0	0	0	–
BOE_060	19,274,440	0	–	0	0	15	0	0	–
BOE_061	17,877,356	0	–	1,150	0	0	0	7	+
BOE_062	18,406,940	0	–	0	0	0	0	0	–
BOE_063	17,469,266	0	–	0	0	0	0	0	–
BOE_064	15,368,202	1	0.4%	0	0	0	0	0	–
BOE_065	19,320,118	20	7.7%	0	0	1	0	1	+
BOE_066	15,492,274	0	–	0	0	0	0	0	–
BOE_067	20,992,354	0	–	0	0	0	0	0	–
BOE_068	18,685,602	0	–	0	0	0	0	0	–
BOE_069	26,924,516	855	5.6%	0	0	0	0	0	–
BOE_070	19,749,830	73,147	95.5%	0	0	0	0	0	–
BOE_073	20,308,534	1	0.4%	0	0	3	0	14,363	+
BOE_074	18,121,652	0	–	2	0	0	0	271	+
BOE_075	6,756,328	0	–	0	0	0	0	174	+
BOE_076	11,615,728	0	–	0	0	0	0	0	–
BOE_077	15,569,022	0	–	0	0	0	0	0	–
BOE_078	26,936,926	1	0.4%	0	0	0	0	0	–
BOE_079	24,925,148	1	0.4%	0	0	0	84	0	–
BOE_084	18,760,962	419	39.1%	0	0	0	0	507	+
BOE_086	18,849,366	0	–	0	0	0	0	0	–
BOE_087	18,183,444	0	–	0	0	0	1	0	–

Figure S1. Standard curve analysis for calculation of EBOV viral loads. A standard curve was generated using an EBOV PCR amplicon spiked into negative plasma matrix across eight 10-fold serial dilutions. The primers and conditions for the PCR assay (qRT-PCR_{US}) are taken from Trombley, et al. [1]. EBOV viral loads are calculated as genome equivalent per milliliter of sample.

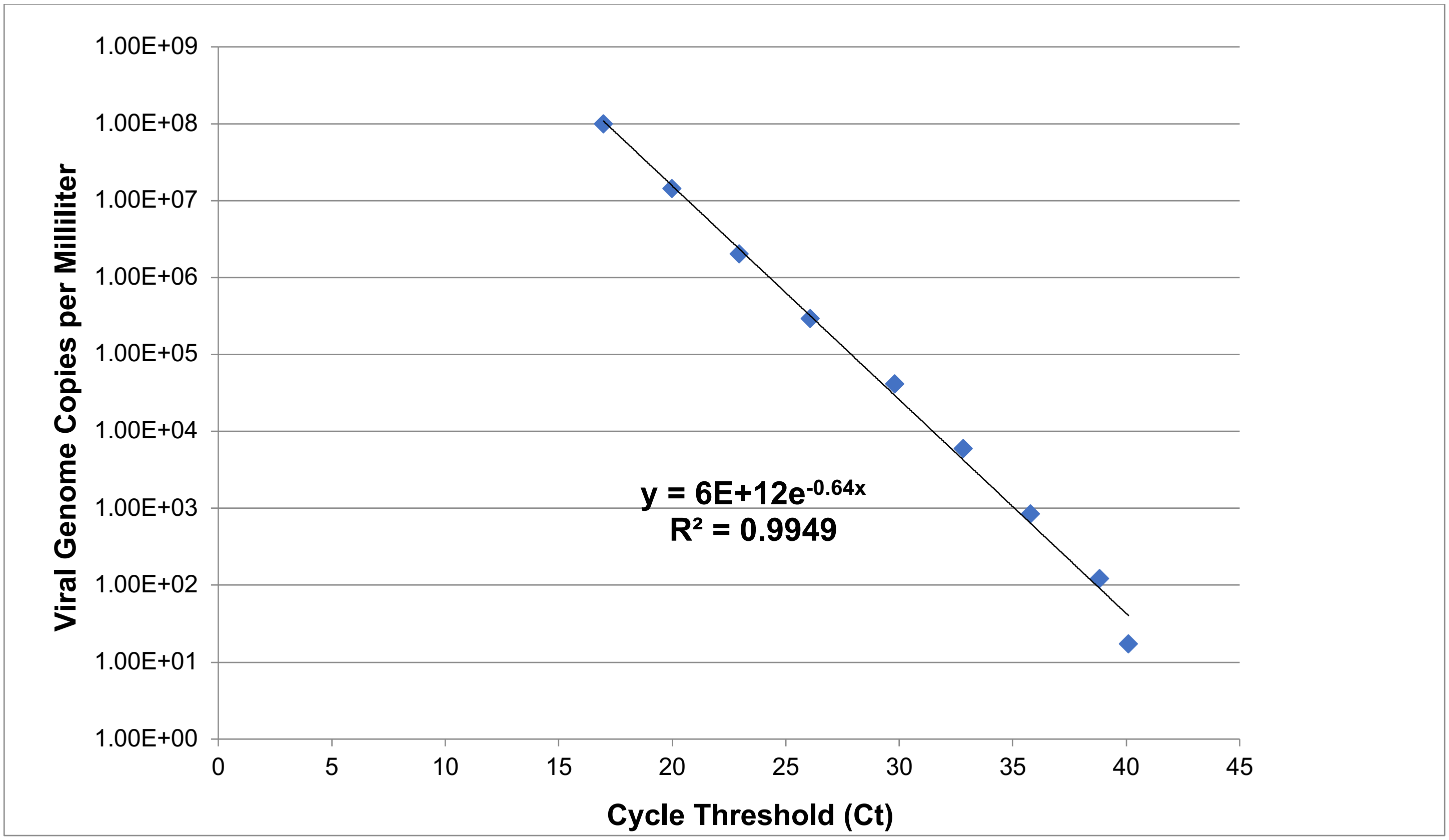

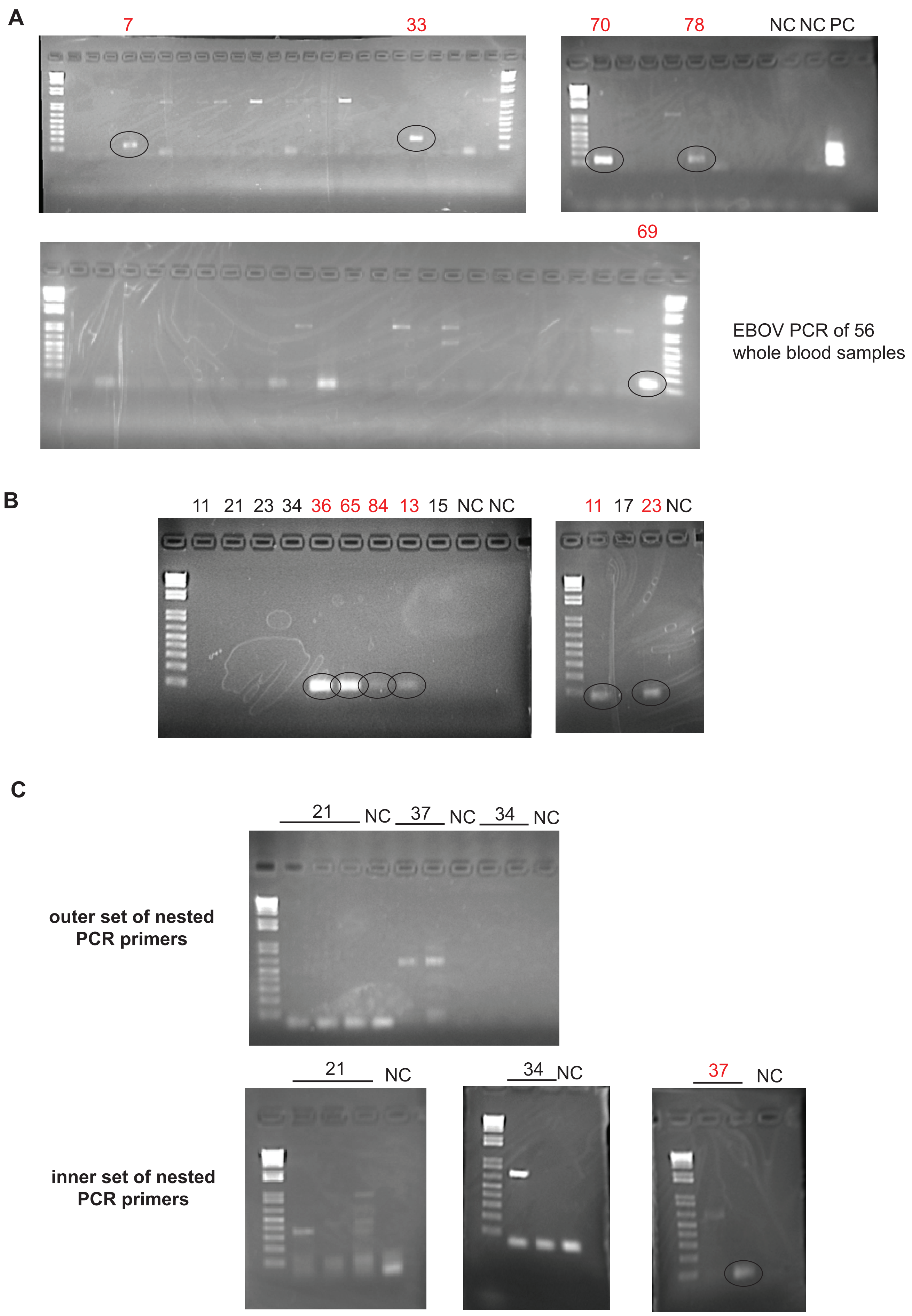
A
7
33
70
78
NC NC PC
69
EBOV PCR of 56 whole blood samples
B
11 21 23 34 36 65 84 13 15 NC NC
11 17 23 NC
C
21
NC
37
NC
34
NC
outer set of nested PCR primers
21
NC
34
NC
37
NC
inner set of nested PCR primers

Figure

Figure S2. Confirmation of EBOV detection by RT-PCR and Sanger sequencing. (A) RT-PCR testing of 56 whole blood samples from suspected EBOV patients (out of 70) with sufficient RNA remaining using the ZEBOV-GP-1F/ZEBOV-0GP-1R primer set targeting the EBOV glycoprotein gene; **(B)** repeat qRT-PCR_{US} testing of whole blood samples from 10 previously PCR-positive EBOV patients using the EBOV assay by Trombley, et al. [1]; **(C)** nested RT-PCR testing of whole blood samples from 3 previously PCR-positive EBOV patients with low viral titers by PCR and mNGS (1 or 2 reads detected), using primers designed from the recovered mNGS reads. Samples containing amplicon bands of expected size (black circles) were Sanger sequenced and confirmed positive for EBOV. The numbers above the lanes refer to the study patient identifier (e.g. BOE_XXX) corresponding to the whole blood sample, with the numbers corresponding to EBOV-confirmed patients by PCR and Sanger sequencing highlighted in red.

Figure S3. Coverage map of *Plasmodium falciparum* reads identified in a single patient. From this patient, we obtained 248,696 reads that aligned to *P. Falciparum* strain 3D (ASM276v2), spanning 12 of the 14 assembled chromosomes.

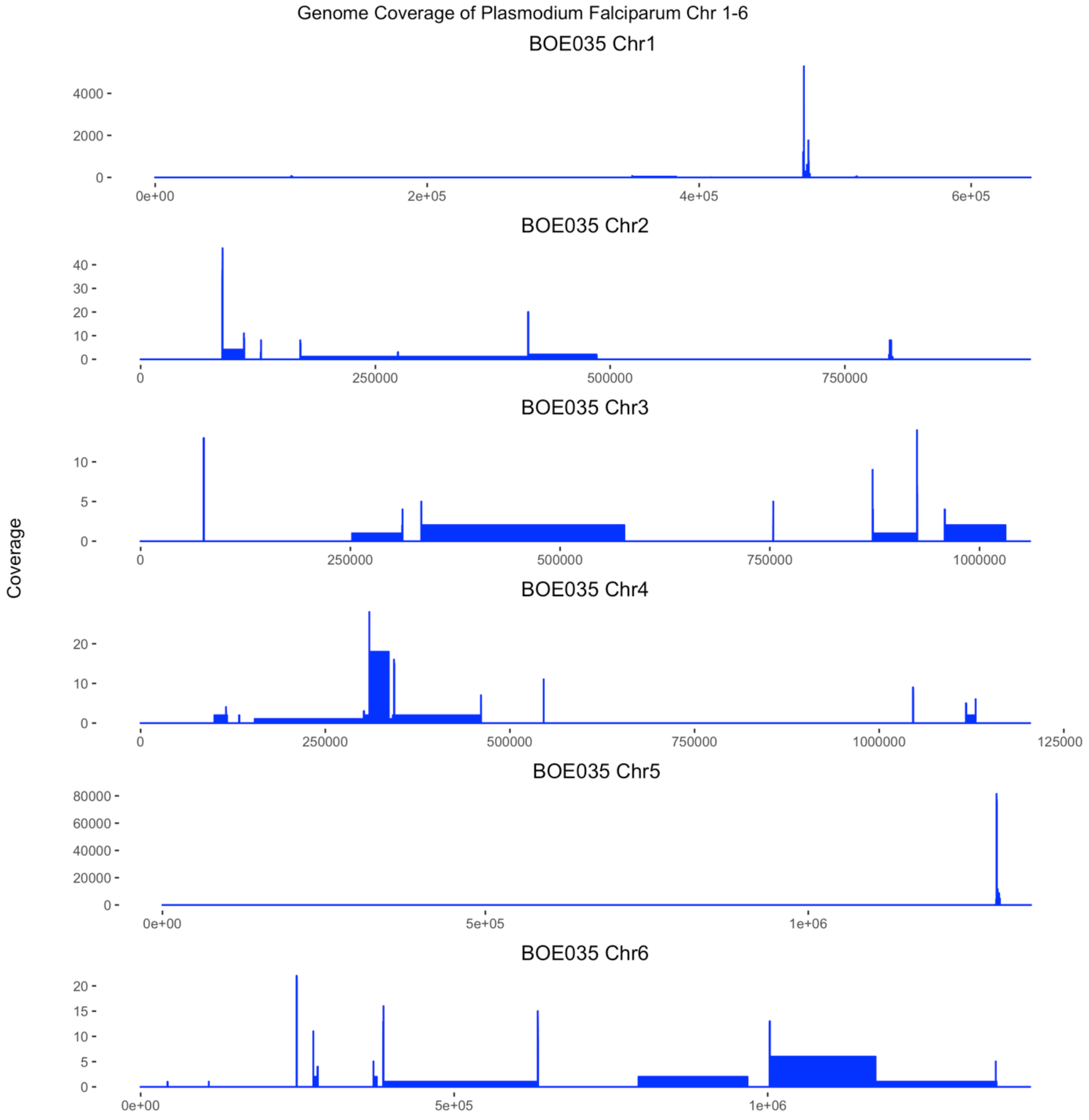

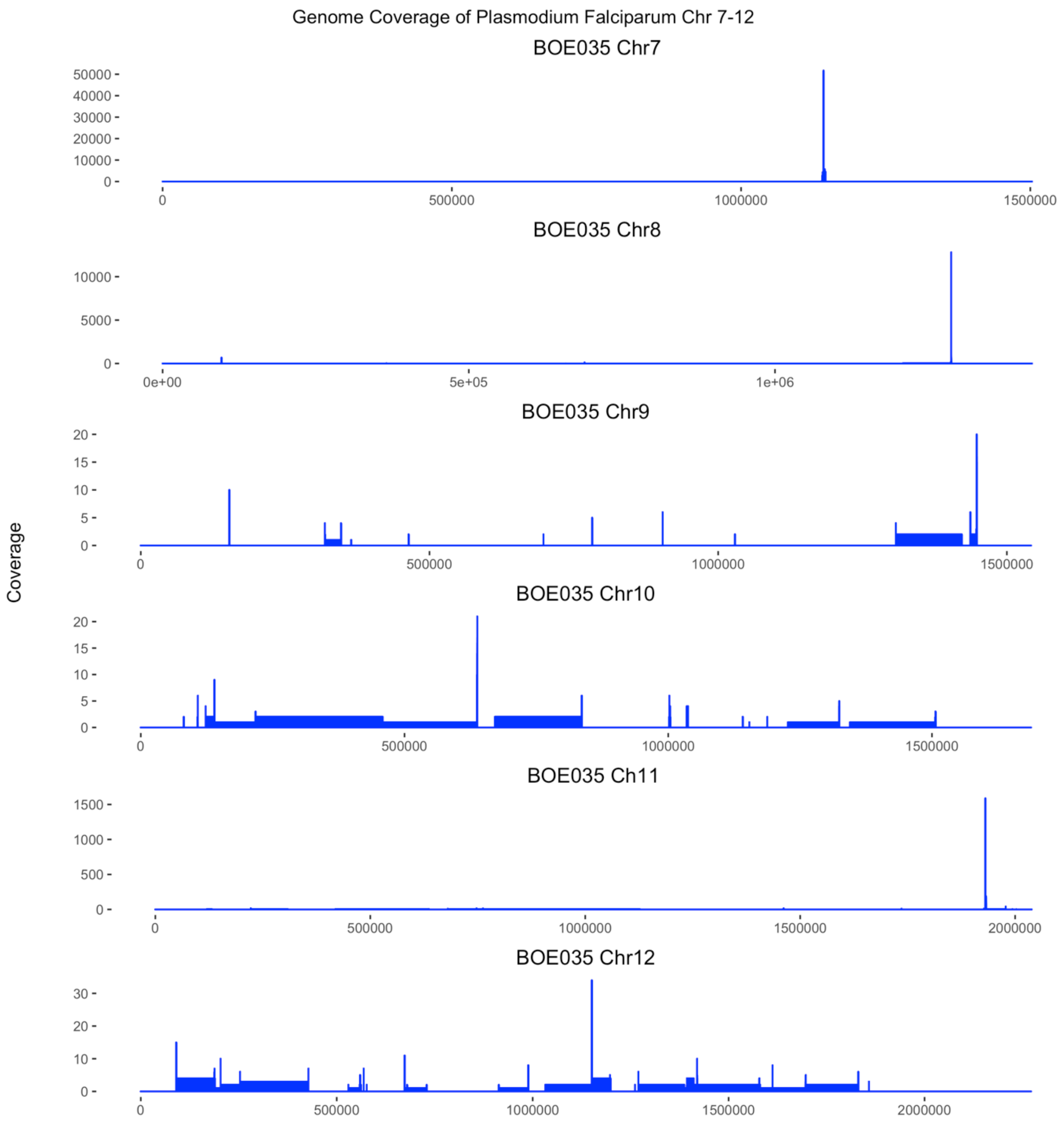

Genome Coverage of Plasmodium Falciparum Chr 7-12
BOE035 Chr7
50000
40000
30000
20000
10000
0
0
500000
1000000
1500000
BOE035 Chr8
10000
5000
0
0e+00
5e+05
1e+06
BOE035 Chr9
20
15
10
5
0
0
500000
1000000
1500000
Coverage
BOE035 Chr10
20
15
10
5
0
0
500000
1000000
1500000
BOE035 Ch11
1500
1000
500
0
0
500000
1000000
1500000
2000000
BOE035 Chr12
30
20
10
0
0
500000
1000000
1500000
2000000

Figure S4. Coverage map of Orungo virus reads identified in a single patient. 84 reads aligned to Orungo virus isolate UGMP 359 (GenBank accession number JQ610676). The plot shows only those segments for which reads were aligned.

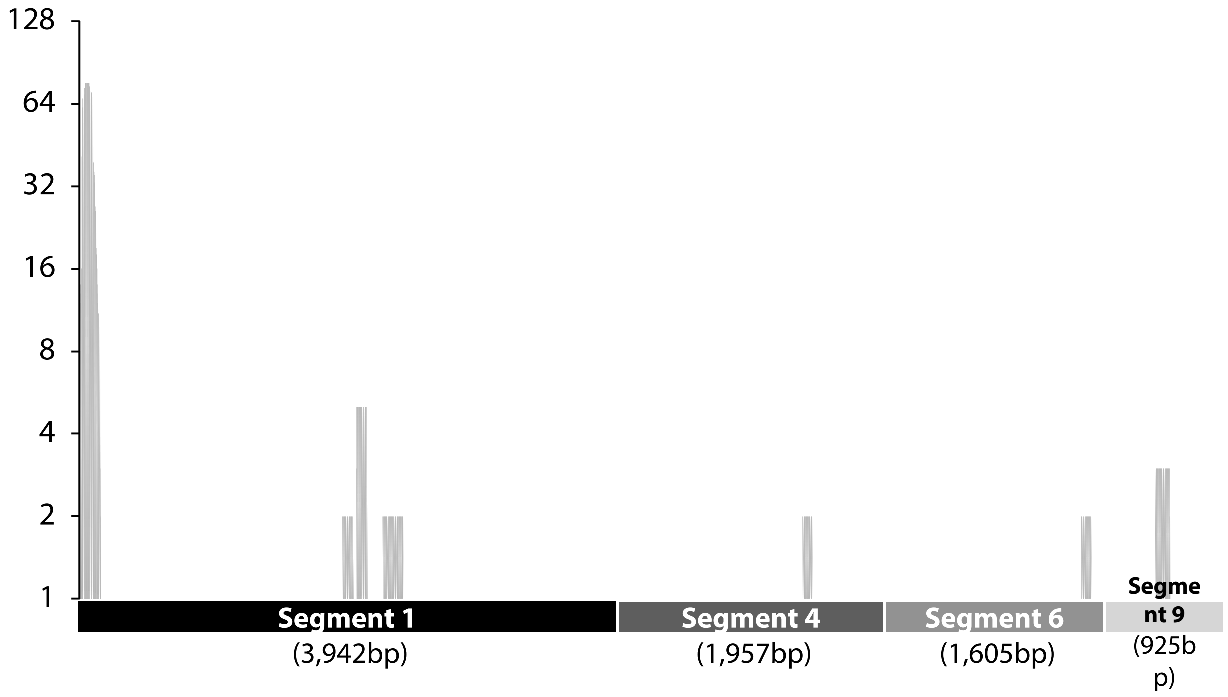

Figure S5. Maximum likelihood phylogeny of the Zaire ebolavirus lineage. The phylogeny was estimated from the complete coding genome sequences of 85 Zaire ebolaviruses, including 19 sequences collected from the 2014 outbreak in the Democratic Republic of the Congo. Node statistical support were assessed using a bootstrap approach (100 replicates). The tree is rooted on the earliest ZEBOV outbreak, the 1976-1977 outbreak in the DRC. The genome sequences generated in this study are shown in bold.

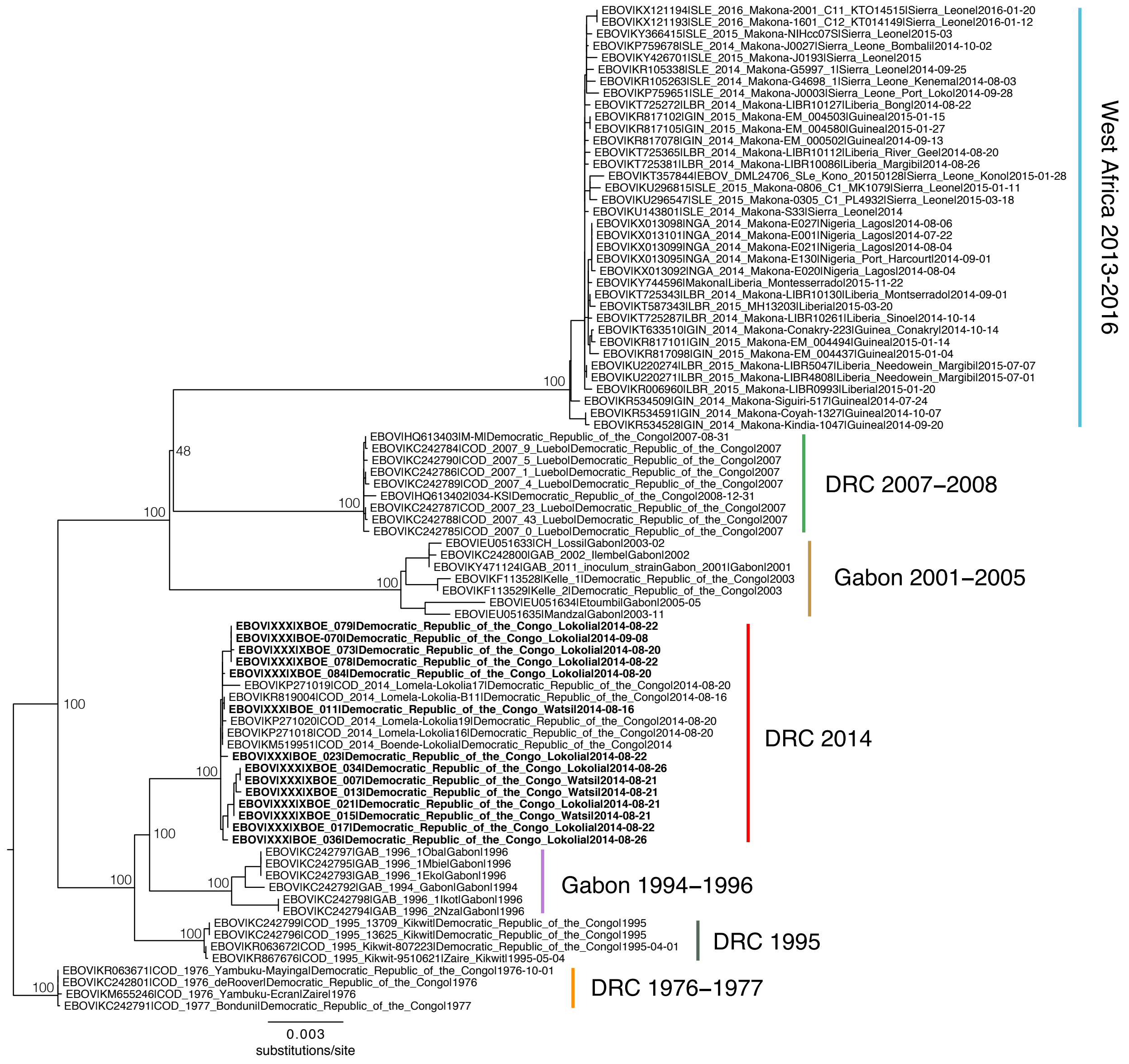

REFERENCES

1. Trombley AR, Wachter L, Garrison J, Buckley-Beason VA, Jahrling J, Hensley LE, Schoepp RJ, Norwood DA, Goba A, Fair JN, Kulesh DA: **Comprehensive panel of real-time TaqMan polymerase chain reaction assays for detection and absolute quantification of filoviruses, arenaviruses, and New World hantaviruses.** *Am J Trop Med Hyg* 2010, **82:**954-960.
